# Identifying the Location of an Accessory Pathway in Pre-Excitation Syndromes Using an Artificial Intelligence-Based Algorithm

**DOI:** 10.3390/jcm10194394

**Published:** 2021-09-26

**Authors:** Thomas Senoner, Bernhard Pfeifer, Fabian Barbieri, Agne Adukauskaite, Wolfgang Dichtl, Axel Bauer, Florian Hintringer

**Affiliations:** 1 University Clinic of Internal Medicine III (Cardiology and Angiology), Medical University Innsbruck, 6020 Innsbruck, Austria; fabian.barbieri@i-med.ac.at (F.B.); agne.adukauskaite@i-med.ac.at (A.A.); wolfgang.dichtl@i-med.ac.at (W.D.); axel.bauer@i-med.ac.at (A.B.); florian.hintringer@tirol-kliniken.at (F.H.); 2 Landesinstitut für Integrierte Versorgung, Tirol Kliniken GmbH, 6020 Innsbruck, Austria; bernhard.pfeier@tiro-kliniken.at; 3 Center for Health and Bioresources, Austrian Institute of Technology, 1210 Vienna, Austria

**Keywords:** Wolff–Parkinson–White syndrome, catheter ablation, artificial intelligence, accessory pathways, algorithms, cardiac electrophysiology

## Abstract

(1) Background: The exact anatomic localization of the accessory pathway (AP) in patients with Wolff–Parkinson–White (WPW) syndrome still relies on an invasive electrophysiologic study, which has its own inherent risks. Determining the AP localization using a 12-lead ECG circumvents this risk but is of limited diagnostic accuracy. We developed and validated an artificial intelligence-based algorithm (location of accessory pathway artificial intelligence (locAP AI)) using a neural network to identify the AP location in WPW syndrome patients based on the delta-wave polarity in the 12-lead ECG. (2) Methods: The study included 357 consecutive WPW syndrome patients who underwent successful catheter ablation at our institution. Delta-wave polarity was assessed by four independent electrophysiologists, unaware of the site of successful catheter ablation. LocAP AI was trained and internally validated in 357 patients to identify the correct AP location among 14 possible locations. The AP location was also determined using three established tree-based, ECG-based algorithms (Arruda, Milstein, and Fitzpatrick), which provide limited resolutions of 10, 5, and 8 AP locations, respectively. (3) Results: LocAP AI identified the correct AP location with an accuracy of 85.7% (95% CI 79.6–90.5, *p* < 0.0001). The algorithms by Arruda, Milstein, and Fitzpatrick yielded a predictive accuracy of 53.2%, 65.6%, and 44.7%, respectively. At comparable resolutions, the locAP AI achieved a predictive accuracy of 95.0%, 94.9%, and 95.6%, respectively (*p* < 0.001 for differences). (4) Conclusions: Our AI-based algorithm provided excellent accuracy in predicting the correct AP location. Remarkably, this accuracy is achieved at an even higher resolution of possible anatomical locations compared to established tree-based algorithms.

## 1. Introduction

Catheter ablation is an established treatment method for accessory pathways (APs) in Wolff–Parkinson–White (WPW) syndrome [1,2,3]. Anatomic localization of the AP prior to catheter ablation is important for pre-procedure planning regarding the access site of the catheter as well as for gauging the risk associated with invasive catheter ablation (especially if the AP is localized in close proximity to the atrioventricular node). Currently, the standard approach for the localization of the AP is the clinician’s interpretation of the 12-lead ECG. However, this method has several limitations, and the localization may differ among clinicians. Thus, the gold standard for localization of the AP in patients with WPW syndrome is the invasive electrophysiologic study (EPS), which has its own inherent risks.

Over time, many surface ECG algorithms for the anatomic localization of the AP have been proposed with varying degrees of accuracy. Three different algorithms have previously been described by Arruda et al. [4], Milstein et al. [5], and Fitzpatrick et al. [6] for determining the localization of the AP based on the pre-excitation pattern during sinus rhythm in the standard surface ECG. However, these algorithms are complicated, time consuming, and only differentiate a limited number of possible anatomic locations. For example, the algorithm by Arruda differentiates between 10 locations, the algorithm by Milstein differentiates between 5 locations, and the algorithm by Fitzpatrick differentiates between 8 locations.

An accurate, rapid, and non-invasive assessment of the correct AP location would therefore be of great clinical value for planning the optimal treatment strategy for the individual patient. Here, we report on an artificial intelligence-based algorithm (locAP AI) using neural networks, which we designed and validated in order to improve and simplify the non-invasive identification of the location of an accessory pathway by means of the 12-lead ECG.

## 2. Materials and Methods

### 2.1. Study Design and Participants

**Figure 1:**
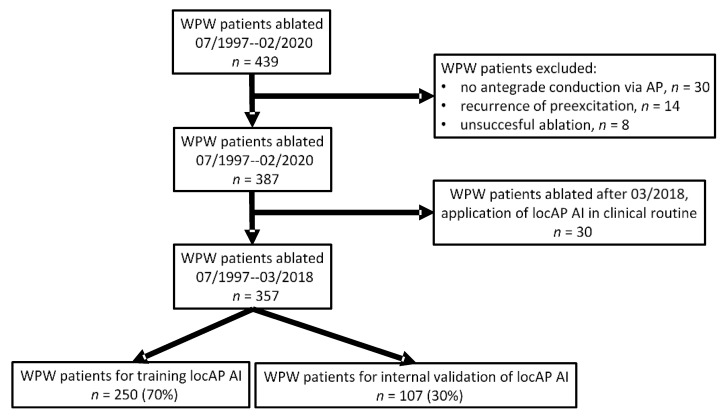
Flow chart of patients included in the present analysis.

This retrospective cohort study included 357 consecutive patients with WPW syndrome who underwent a successful catheter ablation at the Medical University of Innsbruck, Austria, from July 1997 to March 2018. The ethics committee of the Medical University of Innsbruck waved the requirement for informed consent in a general statement concerning retrospective studies. Written informed consent for the catheter ablation procedure was obtained from all patients. Patients with concealed WPW syndrome, those having a recurrence of pre-excitation on the ECG following an initial catheter ablation, and patients with multiple APs or recurrent symptoms were excluded from the analysis (Figure 1).

Four independent electrophysiologists, who were unaware of the results of the electrophysiologic study (EPS), i.e., the successful ablation site, assessed the polarity of the delta wave in all 12 leads of a standard resting ECG registered prior to catheter ablation. The anatomic location of the accessory pathway was defined as the site of successful catheter ablation.

In addition, the AP location was determined using three established tree-based algorithms (Arruda et al. [4], Milstein et al. [5], and Fitzpatrick et al. [6]).

### 2.2. Statistical Analysis 

The predictive accuracy of locAP AI in predicting 1 of the 14 possible AP locations as defined by the EHRA [7] was assessed by sensitivity analysis (sensitivity, specificity, and balanced accuracy) and receiver operating characteristic (ROC) analysis in the total of 357 patients. The predictive accuracy of locAP AI was then compared with the three algorithms proposed by Arruda [4], Milstein [5], and Fitzpatrick [6], as assessed by the chi-square test. Quantitative variables are expressed as means ± standard deviation (SD). Categorical variables are expressed as absolute values and percentages. Statistical analyses were performed by R (version 3.6.3; the R Project, Vienna, Austria).

### 2.3. LocAP AI

For the neural network, a classical network with backpropagation was used. For the activation function, a classical logistic or sigmoidal function with 1/(1-e^-z) was used. We also used a rectified linear unit (ReLU) with less success compared to the logistic function. The maximal number of steps, if the system does not converge, was set to 10e+7 steps. The system consists of 16 input channels, 18 output channels that hold the value with the probability of the location, and 3 hidden layers. The hidden layers have 21 nodes in the first hidden layer, 13 in the middle-hidden layer, and 9 in the third layer. The usage of more than 3 hidden layers did not improve the overall outcome, while a reduction in the hidden layers made the system less efficient in terms of prediction. With a learning rate of 0.2, we achieved the best results among the rates we tested in the system. The tested values were 0.1, 0.2, 0.5, and 0.01. 

Hyperparameters have a great impact on the training of a neural network. Especially computing time, memory consumption, and net accuracy can be influenced. In our case, the training needed about a day until convergence. By training runs with different configurations, an optimal selection of hyper parameters could be found. With this approach that is widely used in the artificial intelligence community, a good setting for our problem could be found.

### 2.4. Validation

The dataset consisting of 357 patients undergoing a catheter ablation from July 1997 to March 2018 was divided in a 70% to 30% fashion. Then, 70% of the dataset was used to train the neural network, while 30% of the dataset, which was never seen by the neural network, was used for an internal validation.

After the training and validation of the neural network, we introduced the locAP AI algorithm into our clinical routine and continued to compare the predicted localization of an AP with the site of its successful ablation.

In a future step, locAP AI will be available as a mobile app in order to broaden its clinical application. The data input from users—pre-excitation pattern and site of successful catheter ablation—will enable us to perform a robust, large-scale, prospective, external validation.

## 3. Results

Table 1 shows the baseline characteristics of the 357 study patients. 

The mean age at ablation was 34.3 ± 15 years (range 7–78 years), and 63.9% of patients were male. The mean QRS width was 121 ± 21 ms, while the mean PR interval was 114 ± 21 ms. The study population included 15.4% patients younger than 18 years of age. In total, 39.2% of patients had a QRS width ≥ 120 ms and a PR interval ≤ 120 ms. Among these patients, 19.3% were below the age of 18 at the time of ablation. Table 2 depicts the abbreviations used in this report to describe the various AP locations.

The frequency (the number of observed cases) of each AP location is shown in Table 3.

The most frequent AP locations were, in descending frequency, inferoparaseptal, left superoposterior, and left posterior (Table 3).

LocAP AI achieved a predictive accuracy of correctly identifying the AP location of 85.7% (95% CI 79.6–90.5, *p* < 0.0001) (Table 4).

**Figure 2:**
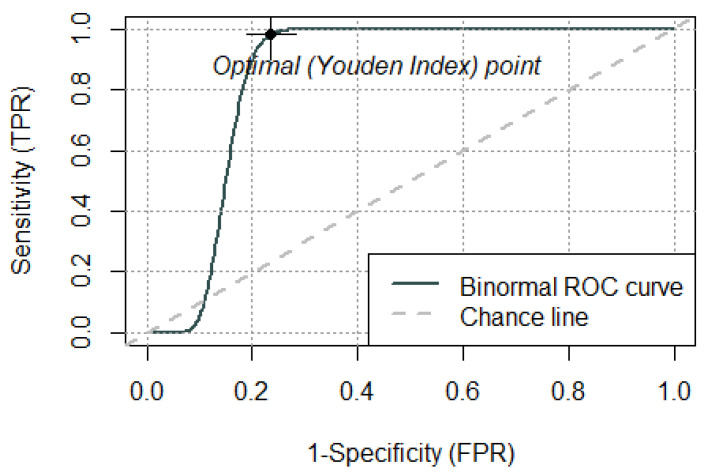
Receiver operating characteristic analysis yielded an area under the curve of 0.847.

A ROC analysis yielded an area under the curve of 0.847 (Figure 2).

The established algorithms proposed by Arruda [4], Milstein [5], and Fitzpatrick [6] achieved predictive accuracies of 53.2%, 65.6%, and 44.7%, respectively. At lower anatomical resolutions identical with these three algorithms, locAP AI achieved a predictive accuracy of 95.0%, 94.9%, and 95.6%, respectively (*p* < 0.001 for all).

As shown in Table 4, the predictive accuracy of locAP AI was high for frequent AP locations but only modest for rare AP locations.

## 4. Discussion

### 4.1. Artificial Intelligence—Neural Network

Artificial intelligence (AI) refers to the attempt to mimic certain human decision-making structures. Furthermore, when using the term artificial intelligence, two types are defined and used. Firstly, strong AI tries to generate a human-like, acting intelligent system, which is still very visionary. Secondly, weak AI is used to solve concrete application problems with high accuracy. For our specific problem, a classical neural network consisting of several linked neurons with implemented state transition functions was used. The neurons are also called units or nodes. These elements collect information from the environment (input parameters) or from other neurons (hidden layers) and are connected to each other by edges. The strength of the connection between two neurons is expressed by a weight. The greater the absolute value of the weight, the greater the influence of one unit on another unit.

A positive weight indicates that one neuron exerts an excitatory influence on another neuron. A negative weight means that the influence is inhibitory. A weight of zero means that one neuron currently has no influence on another neuron. Therefore, the complete knowledge of a neural network is stored in its weights. Learning is usually defined in such a system as weight changes between units. Furthermore, so-called bias units with no input but having an activity level of one are commonly used. If the input from the units is very low, then the bias unit guarantees that the positive weight remains active. 

With neural networks, a differentiation between the training phase and the propagation phase is made. In the training phase, the neural network learns by means of the given learning material. Accordingly, the weights between the individual neurons are changed according to the used learning rules.

### 4.2. LocAP AI

Our study demonstrates that locAP AI provides excellent accuracy in predicting the anatomic correct AP location. Remarkably, this accuracy is achieved at an even higher resolution with 14 anatomical locations compared to the classical tree-based algorithms [4,5,6], which are most commonly used in clinical practice. The aforementioned algorithms use a tree-based algorithm and, as such, have their limitations in a clinical setting, as they are potentially complicated and time consuming to apply. To overcome these limitations, in 2012, our group developed an algorithm called locAP, which is based on a score [8]. The inventive step of the algorithm described in this paper is the application of AI by using a self-learning neural network.

Even though WPW ablation has generally been viewed to be safe and effective, approximately 6% of ablations are still unsuccessful [9]. The exact localization of the AP is crucial for the success of catheter ablation. The exact localization of the AP prior to catheter ablation using an inexpensive, widely available, point-of-care test—the ECG—provides the electrophysiologist with important information regarding the proximity of the AP to the normal conduction system and the subsequent risk of AV block associated with an ablation attempt, as well as the need for left heart catheterization and transseptal atrial access and their potential complications. Furthermore, it may help in the planning of the ablation procedure, such as the use of cryoablation for septal APs in close vicinity to the AV node. Therefore, a non-invasive assessment of AP localization might be most advantageous in cases of high-risk AP locations.

Tree-based algorithms, such as those we applied in our study population in order to compare the performance of the locAP AI algorithm, have several limitations. They are unstable, meaning that a small change in the data can lead to a large change in the structure of the optimal decision tree. Furthermore, for data including categorical variables with different number of levels, information gain in decision trees is biased in favor of those attributes with more levels [10]. In studies that tested these algorithms, their accuracy was even lower than that previously reported by their designers (predictive accuracy of around 70%) [11]. Therefore, these algorithms can be regarded as orientation only prior to catheter ablation. These limitations can be overcome using AI-based algorithms, which are not as susceptible to small changes as tree-based algorithms are. Furthermore, AI-based algorithms, especially in the context of machine learning, are capable of automatic improvement through experience without the help of a human [12].

After the training and validation of the neural network, we have introduced the locAP AI algorithm into our clinical routine and continuously compared the predicted localization of an AP with the site of its successful ablation. Due to the limited number of cases in our clinical routine to date, at this point in time, we are not able to perform a robust prospective validation. However, observation of our clinical routine revealed an accuracy of 77%. We observed failures either at the right free wall, an area where APs are less frequent, or in the septum. In all cases where locAP AI failed, it still located the AP to a site directly next to the site of successful catheter ablation.

We will make locAP AI available as a mobile app in order to broaden its clinical application. The data input from users—pre-excitation pattern and site of successful catheter ablation—will enable us to perform a large-scale, prospective, external validation.

### 4.3. Limitations

Even an algorithm such as the locAP AI algorithm is limited by variability in anatomy (e.g., rotation of the heart within the thorax), variable degree of pre-excitation and QRS fusion, the presence of more than one manifest accessory pathway, intrinsic ECG abnormalities (such as prior myocardial infarction or ventricular hypertrophy), patient body habitus, and technical variability in ECG acquisition and electrode positioning [13]. Although we have made every effort to reduce bias, such as blinding of ECG analyses and internal validation, the algorithm still needs to be validated in independent cohorts.

In our analysis, some locations had a reduced accuracy of the locAP AI algorithm, such as PH, which is a critical AP location with respect to potential damage to the AV node during ablation. This limitation might reduce the aim of risk stratification prior to catheter ablation. Furthermore, most AP locations with reduced accuracy are located on the right side, which is a location known to be the most difficult to be ablated effectively.

## 5. Conclusions

Our AI-enabled ECG algorithm for the prediction of the location of the AP in WPW syndrome is able to correctly identify the AP location with high accuracy. The locAP AI algorithm has important implications for the management of such patients regarding the planning of the ablation as well as in preoperative risk prediction.

## Figures and Tables

**Table 1:** Baseline characteristics of the study population.

Variables	Study Population (*n* = 357)
Male, *n* (%)	228 (63.9)
Age, y, mean ± SD	34.3 ± 15
QRS duration, ms, mean ± SD	121 ± 21
PR interval, ms, mean ± SD	114 ± 21
Patients < 18 y of age, *n* (%)	55 (15.4)
QRS duration ≥ 120 ms and PR interval ≤ 120 ms, *n* (%)	140 (39.2)

MS: milliseconds; SD: standard deviation; Y: years.

**Table 2:** Abbreviations used in this report to describe the different bypass tract locations.

Abbreviation	Attitudinally Correct	Attitudinally Incorrect
RS	Right superior	Right anterior
RSA	Right superoanterior	Right anterolateral
RA	Right anterior	Right lateral
RIA	Right inferoanterior	Right posterolateral
RI	Right inferior	Right posterior
LS	Left superior	Left anterior
LSP	Left superoposterior	Left anterolateral
LP	Left posterior	Left lateral
LIP	Left inferoposterior	Left posterolateral
LI	Left inferior	Left posterior
SPS	Superoparaseptal	Anteroseptal
IPS	Inferoparaseptal	Posteroseptal
S	Septal	Midseptal
PH	Parahisian	

**Table 3:** The frequency and percentage of each bypass tract location (*n* = 357).

Location	Frequency	Percentage (%)
IPS	81	22.70
LSP	71	19.89
LP	70	19.61
S	32	8.96
LIP	26	7.28
LI	23	6.44
SPS	15	4.20
RI	8	2.24
RA	8	2.24
RS	7	1.96
RSA	5	1.40
PH	4	1.12
LS	4	1.12
RIA	3	0.84
Total	357	100.00

**Table 4:** Sensitivity analysis for the accurate prediction of the accessory pathway by the locAP AI algorithm for each of the 14 different pathways.

	1	2	3	4	5	6	7	8	9	10	11	12	13	14
AP	IPS	LSP	LP	S	LIP	LI	SPS	RI	RA	RS	RSA	PH	LS	RIA
Total	81	71	70	32	26	23	15	8	8	7	5	4	4	3
Sensitivity	0.9706	1.0000	0.9714	1.0000	0.69231	1.00000	0.85714	0.00000	1.00000	1.00000	0.00000	0.00000	0.00000	0.0000
Specificity	1.0000	0.9933	0.9929	1.0000	0.98765	0.99394	1.00000	1.00000	0.88304	1.00000	1.00000	1.00000	1.00000	1.0000
Prevalence	0.1943	0.1429	0.2000	0.1200	0.07429	0.05714	0.04000	0.02857	0.02286	0.01714	0.01143	0.01714	0.01143	0.0000
Balanced Accuracy	0.9853	0.9967	0.9821	1.0000	0.83998	0.99697	0.92857	0.50000	0.94152	1.00000	0.50000	0.50000	0.50000	0.5000

## Data Availability

Data are available upon reasonable request.
